# Predicting peptide orientation in membrane interactions: a review on transmembrane and surface-bound states

**DOI:** 10.1007/s00249-025-01810-7

**Published:** 2026-01-23

**Authors:** Lúcio Otávio Nunes

**Affiliations:** Directory of Research, Federal University of Jequitinhonha and Mucuri Valleys, MGT 367 Road, Km 583, n. 5000, Alto da Jacuba, Diamantina, Minas Gerais 39100-000 Brazil

**Keywords:** Antimicrobial peptides, Membrane interaction, Surface-bound state, Transmembrane insertion

## Abstract

Understanding the mechanism of action of antimicrobial peptides (AMPs) is a key step in developing new antimicrobial agents. This mechanism is often linked to the peptide’s orientation upon interaction with lipid membranes—whether it inserts into the membrane or remains surface-bound. Despite its importance, the literature lacks a systematic approach to predict peptide orientation based on physicochemical characteristics. This review highlights how peptide orientation at membranes can be qualitatively predicted by integrating sequence-level features, length/effective span, amphipathicity, charge distribution, and helix propensity, with membrane/environmental variables. The approach is illustrated with representative AMPs and it is discussed how external factors (pH, anionic fraction, cholesterol, ionic strength) bias surface-bound versus transmembrane regimes. The review is built upon theoretical observations and integrates structural, thermodynamic, and electrostatic parameters drawn from experimental data in the literature. While this study does not aim to provide a definitive classification, it offers a starting point that may guide experimental validation and future refinement.

## Introduction

Antimicrobial peptides (AMPs) are molecules found in a wide variety of living organisms, including humans, animals, fungi, and even bacteria. They are part of the innate immune system and represent the first line of defence against pathogens. Given their natural role and the increasing concern over antibiotic resistance, AMPs have emerged as promising candidates for the development of new antibacterial drugs, particularly because their mechanisms of action differ significantly from those of conventional antibiotics. Most AMPs act by targeting bacterial membranes, which are broad and nonspecific structures. This mode of action reduces the likelihood of resistance development. Therefore, since AMPs primarily act at the membrane level, understanding peptide–membrane interactions becomes essential for both fundamental science and drug design (Zasloff 2002).

Despite their biological relevance, the mechanisms of action of antimicrobial peptides (AMPs) are not yet fully understood. Some peptides insert into the bacterial membrane, others associate in a pore-forming manner, and still others interact only with the membrane surface. Because of this diversity, it is extremely important to determine how each peptide interacts with the membrane, especially in the context of designing peptides that are both more active and less toxic to human cells (Wimley 2010).

The difficulty in identifying the mode of interaction arises from the complex nature of peptide–membrane interactions. These are multifactorial processes, governed primarily by the physicochemical properties of both the peptide and the membrane. Understanding the possible orientation of a peptide—whether surface-bound or transmembrane—based on these parameters can be highly valuable for rational design and functional prediction (Ladokhin and White 1999).

Since understanding the mechanism of action of antimicrobial peptides is crucial, determining whether a given peptide interacts in a transmembrane or surface-bound manner is of great value. However, this information is not readily available for all peptides, due to the complexity of the factors involved in peptide–membrane interactions, as previously mentioned. The current literature is largely composed of isolated studies, each analysing different physicochemical properties in different peptides and experimental contexts. As a result, there is still no unified study capable of predicting whether a peptide will associate with the membrane via insertion or surface binding (Bechinger 1997).

The goal of this review is to organise, in a concise framework, the factors that bias peptide orientation (surface-bound, transmembrane, or partially inserted). The review synthesises structural, thermodynamic, and electrostatic evidence into a decision map to guide hypothesis generation and experimental validation, it does not claim quantitative classification.

## Theoretical considerations

Although the mechanism of action of antimicrobial peptides is not fully understood, several classical frameworks have been proposed and are widely accepted in the literature. The first is the barrel-stave model, in which amphipathic helical peptides insert perpendicularly into the lipid bilayer. The hydrophobic face of each helix aligns with the membrane’s hydrophobic core, while the hydrophilic faces collectively form the inner lining of a pore, creating a transmembrane channel resembling a barrel made of peptide “staves”. The second is the toroidal pore model, where peptide insertion induces a curvature in the membrane such that the lipid monolayers bend continuously through the pore. In this structure, both the peptides and the phospholipid headgroups line the interior, forming a pore stabilized by electrostatic and hydrogen-bond interactions between the peptides’ hydrophilic faces and the lipid heads. Lastly, in the carpet model, multiple peptides bind parallel to the membrane surface, covering it like a carpet. When a threshold concentration is reached, the peptides destabilize the membrane in a detergent-like manner, leading to micellization and membrane disruption without forming defined pores (Wimley 2010).

It is well established that peptide–membrane interactions follow an organized and non-random pattern. Fundamentally, such interactions occur only when the overall Gibbs free energy change (ΔG) is negative, making the process thermodynamically favourable. One major contribution to this energy change is electrostatic attraction: cationic peptides are drawn to the negatively charged surfaces of bacterial membranes. This attraction typically lowers the system’s electrostatic potential and is frequently accompanied by heat release, characterizing an exothermic, enthalpically driven process (ΔH < 0). Additionally, both the phospholipid headgroups and the peptide molecules are initially surrounded by structured hydration shells. Upon interaction, many of these water molecules are displaced and released into the bulk solvent, increasing the system’s entropy (ΔS > 0). This gain in entropy further contributes to the spontaneity of the peptide–membrane association (Seelig 2004).

In addition to thermodynamic principles, several structural features of antimicrobial peptides play a critical role in modulating peptide–membrane interactions. One of these is helical content. It is well established that upon dehydration by surrounding water molecules, peptide backbones tend to adopt a helical conformation, which stabilizes the structure and facilitates membrane association. Another important feature is amphipathicity. Since the lipid bilayer presents an amphipathic environment—with both hydrophobic cores and polar headgroups—peptides displaying amphipathic character can align favourably with the membrane, often contributing to selective and effective binding. Peptide length is also influential. Longer peptides are more likely to span the membrane and engage in transmembrane interactions, whereas shorter peptides typically associate at the surface. Finally, orientation—whether the peptide aligns parallel or perpendicular to the bilayer—strongly affects its mechanism of action, influencing whether it disrupts the membrane surface or inserts to form pores (Vishnepolsky and Pirtskhalava 2014).

Biological membranes and their surrounding milieu are not passive backdrops; they are responsive elements that modulate peptide orientation upon membrane encounter. Accordingly, additional context variables can be formalized as parameters that govern peptide–membrane interactions and should be made explicit. These variables include membrane lipid composition, cholesterol fraction, surface charge density, and hydrophobic thickness; as well as environmental conditions—pH, ionic strength, and the resulting protonation states of both the peptide and the lipid headgroups—which together tune adsorption, interfacial anchoring, and hydrophobic-mismatch penalties that bias surface-bound versus transmembrane states (Sanderson 2005; Sani and Separovic 2016).

We next outline the context variables that modulate orientation (composition, thickness, pH, ionic strength, cholesterol). These variables act in the process that will culminate in the biological action of the peptide; they are strictly related to the peptide’s route of action.

**Fig. 1:**
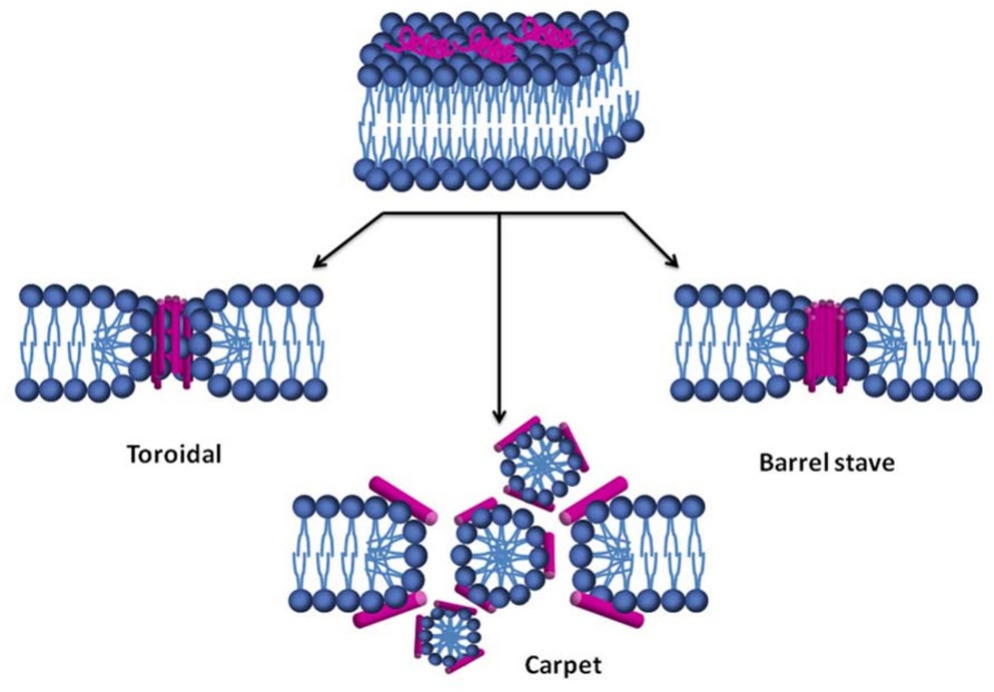
Schematic view of the three most described mechanisms of action for antimicrobial peptides, toroidal pore, barrel-stave and carpet. *Source*: (Silva et al. 2014). This file is licensed under the Creative Commons Attribution 3.0 Unported license.

There were described before three canonical routes, barrel-stave, toroidal, and carpet/micellization, highlighting lipid participation and ordering requirements. We emphasise that persistent, protein-lined pores (barrel-stave) are special cases demanding low electrostatic repulsion and high oligomeric order. In contrast, most membrane activities arise from interfacial activity that transiently recruits lipids (toroidal) or disrupts packing without a discrete pore (carpet). For reference, Fig. 1 summarises the three canonical permeabilisation routes, barrel-stave, toroidal, and carpet/micellization, highlighting lipid participation and ordering requirements.

**Fig. 2:**
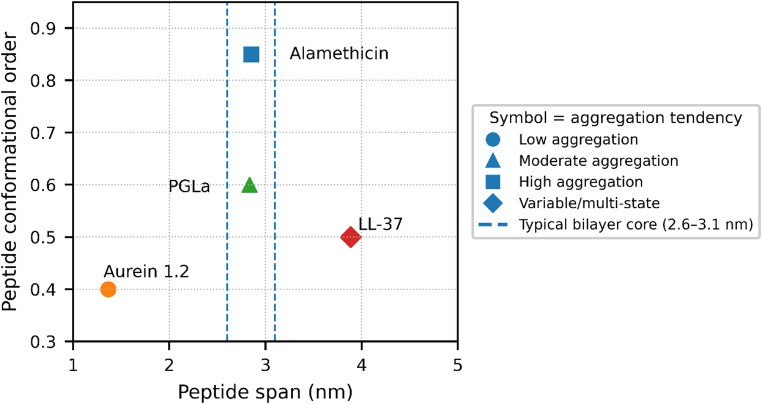
Mapping peptide orientation: effective span versus conformational order

Figure 1 serves as a didactic overview used throughout the review for context; orientation-focused reasoning is developed in the decision map of Fig. 2.

## Parameters governing Peptide–Membrane interactions

### Structural parameters

#### Peptide length and membrane thickness

It is well known that different microorganisms exhibit membranes with varying properties, one of which is membrane thickness. Some bacterial species have relatively thin membranes, while others present thicker bilayers, and this variation significantly influences peptide interaction. On the other hand, the length of the peptide also plays a crucial role. Short peptides may not be able to span the membrane, whereas longer ones may favour transmembrane orientations. Therefore, to understand peptide–membrane interactions more accurately, it is essential to consider both peptide length and membrane thickness simultaneously (Grage et al. 2016).

#### Helical propensity

Another key structural parameter is the peptide’s propensity to adopt an alpha-helical conformation. In many cases, peptides fold into helices upon interaction with membranes, particularly after dehydration of the peptide backbone. This structural organization often facilitates insertion or stable binding. However, not all peptides achieve helical conformations, and some may remain unstructured or adopt other secondary structures depending on their sequence and environment (Biggin and Sansom 1999).

#### Amphipathicity and transverse dipole moment

Amphipathicity is also one feature that influences peptide-membrane interaction. As the lipid bilayer is inherently amphipathic—with hydrophilic headgroups and hydrophobic cores—peptides that also display amphipathic character can align favourably with the membrane interface. Amphipathic helices tend to associate more effectively with membranes than unstructured or non-amphipathic peptides. Additionally, the distribution of hydrophobic and hydrophilic residues along the helix gives rise to a hydrophobic moment (a form of transverse dipole), which enhances orientation and anchoring within the membrane environment (Wieprecht et al. 1997).

#### Side chain steric hindrance and residue packing

The steric hindrance of amino acid side chains is another parameter that modulates peptide–membrane interaction. Smaller residues such as glycine and alanine favour tight packing and membrane insertion. In contrast, bulky aromatic residues like tryptophan, while highly hydrophobic, may disrupt packing due to their large volume. These differences influence the way peptides adjust spatially to the lipid environment and affect the efficiency and mode of insertion or surface alignment (Quint et al. 2010).

### Electrostatic and hydrophobic factors

#### Net charge and charge distribution

Since the initial interaction between peptides and membranes is primarily driven by electrostatic attraction, the net charge of the peptide is a crucial parameter. Positively charged peptides are attracted to the negatively charged phosphate groups present on bacterial membranes, promoting initial membrane binding. Moreover, the distribution of charges along the peptide sequence may also influence the orientation and stability of binding, as localized clusters of positive charges can facilitate stronger and more directed interactions with the membrane surface (Yin et al. 2012).

#### Aromatic residues at interfacial positions

Another important factor is the positioning of aromatic residues, such as tryptophan (Trp), tyrosine (Tyr), and phenylalanine (Phe), along the peptide sequence. While these residues are highly hydrophobic, their bulky side chains may disrupt lipid packing if located deep within the membrane core or in central positions of the peptide. However, when positioned near the terminal regions or at interfacial sites, aromatic residues can stabilise peptide–membrane binding through interactions with lipid headgroups, including cation–π interactions or anchoring effects at the membrane interface (Johnson et al. 2007).

#### Global hydrophobicity and residue localisation

The overall hydrophobicity of a peptide must exceed a certain threshold to enable insertion or strong association with the membrane’s hydrophobic core. Nonetheless, not only the quantity but also the distribution of hydrophobic residues is critical. Peptides that present hydrophobic residues concentrated on one face of the molecule—i.e., that display a hydrophilic/hydrophobic partitioning—tend to interact more effectively with the amphipathic nature of lipid bilayers. This alignment favours stable membrane orientation and can enhance selectivity and efficiency of interaction (Yin et al. 2012).

#### Arginine-specific interactions

Among the positively charged amino acid residues, arginine has a particular action. Arginine’s guanidinium can form bidentate hydrogen bonds and ion pairing with phosphate/ester oxygens while dragging water and headgroups into the interface. This stabilises penetration of positively charged segments more effectively than lysine and helps explain why Arg-rich peptides bind and remodel membranes strongly. In the design of peptides, distributing Arg rather than clustering can reduce peptide–peptide repulsion while maintaining strong interfacial anchoring (Tang et al. 2007, 2008).

#### Anionic antimicrobial peptides and electrostatics

Although most AMPs are cationic, a subset is anionic and operates under different electrostatic constraints. On anionic membranes initial adsorption is weak without counterions; activity often relies on divalent-cation bridging (Zn²⁺, Ca²⁺, Mg²⁺) that screens charge and coordinates peptide carboxylates to lipid phosphates. In dermcidin (DCD-1 L, an anionic peptide), metal binding also promotes channel-like oligomers. pH tunes these effects via protonation of Asp/Glu and termini, increasing interfacial affinity at lower pH and shifting surface↔inserted equilibria. Beyond net charge, amphipathicity, helix propensity, and effective span remain decisive (Becucci et al.^2014^; Zeth and Sancho-Vaello 2017).

### Dynamical and Concentration-Dependent effects

#### Effective concentration and aggregation

An interesting and initially evident aspect of peptide–membrane interaction is the role of concentration. It is somewhat obvious that higher peptide concentrations tend to result in stronger interactions with the membrane. However, when considering peptides that aggregate, this relationship is not entirely linear. If two peptides are compared at the same nominal concentration—one that aggregates and one that does not—there is a subtle but important difference in their effective concentrations. A closer inspection reveals that the effective concentration is higher for the non-aggregating peptide. Thus, this property also plays a significant role in governing the interaction (Singh, et al. 2008).

#### Conformational dynamics

Another important factor is the conformational dynamics of the peptide. These dynamics influence the type of transient states that the peptide adopts during interaction with the membrane. Peptides with high conformational flexibility tend to form fewer stable structures. In contrast, peptides with more restricted dynamics can adopt more organized architectures, such as pore-like structures (Grage et al. 2016).

#### Peptide Cooperation and leakage kinetics

When the kinetics of fluorophore release from vesicles are analysed experimentally, the results can provide valuable—but limited—insights into a peptide’s mechanism of action. A fluorophore such as calcein is small and readily passes through transient defects, the dye itself can perturb bilayer packing at the high intravesicular concentrations used for self-quenching and may weakly interact with cationic peptides, potentially promoting leakage independent of stable pore formation, however, the calcein release is not the unique proof of pore existence (Dutta et al. 2020; Malanovic et al. 2020). An approximately constant rate of fluorophore release suggests persistent conductive pathways, whereas time-dependent rates with burst-like events point to defect accumulation and catastrophic failure consistent with interfacial activity. Distinguishing graded versus all-or-none leakage modes is particularly informative for this purpose (White 1995; Wheaten et al. 2013).

An important point is that dye release depends strongly on membrane composition: differences between neutral and anionic bilayers, as well as changes with ionic strength, can reflect adsorption/binding rather than insertion per se; orthogonal structural data like peptide orientation/tilt by ssNMR helps resolve the mechanism (Ramamoorthy et al. 2006). Another point is that cholesterol often reduces dye release by stabilizing and ordering the bilayer; in such cases, lower leakage can reflect reduced peptide binding and a shift toward interfacial action rather than long-lived pores (Apellániz et al. 2010).

It is important note that the use dye-release as a screen, not as sole mechanistic proof; confident assignments require convergence between kinetics (leakage mode), structure (orientation/tilt), and elasticity/curvature readouts (Wimley 2010).

### Membrane and environment variables

#### Membrane lipid composition and charge

The composition of the membrane is an important feature that modulates the peptide orientation in the interaction (Salnikov et al. 2025). Increasing the mole fraction of anionic lipids with PG, PS or cardiolipin strengthens the initial electrostatic attraction and increases the surface activity of cationic AMPs, biasing toward surface-bound states at low peptide-to-lipid ratios. This enrichment at the interface can be large enough to mask moderate hydrophobic driving forces for insertion. Conversely, predominantly zwitterionic matrices (PC, PE) reduce interfacial binding and may favour insertion when peptide length and amphipathicity are sufficient. Composition also tunes domain formation and line tension, thereby modulating the energetic cost of lipid-assisted pores (Wieprecht et al. 2000; Bechinger 2015; Nagao et al. 2015).

#### Lipid saturation and membrane thickness

Regarding other membrane characteristics, saturated chains like DPPC or DPPE thicken and stiffen bilayers relative to unsaturated ones as POPC or POPG, increasing the hydrophobic span required for transmembrane alignment. This shifts transmembrane-favouring thresholds and can flip in borderline cases like PGLa, between in-plane and inserted states. Hydrophobic mismatch can be mitigated by tilt, local thinning/thickening, or oligomerisation, but these adaptations carry penalties that must be offset by other parameters (Strandberg et al. 2012; Harmouche and Bechinger 2018).

#### Environment pH and peptide/membrane protonation

Some extrinsic variables also interfere with the peptide orientation when it interacts with the membrane. pH modulates both peptide protonation (especially histidine-rich sequences and termini) and, in specific cases, lipid ionization. Protonation of His and acidic termini at lower pH increases cationic charge density and interfacial affinity, stabilizing surface-bound states; deprotonation at higher pH can reduce intra-bundle repulsion and lower the cost of insertion. Reported surface↔transmembrane transitions in His-containing peptides provide quantitative markers for how far decision boundaries shift with ΔpH. While some acidic lipids like phosphatidic acid exhibit appreciable changes in ionization with pH, many anionic lipids, such PG and PS, remain largely deprotonated (negatively charged) over physiological ranges; thus, peptide protonation is often the dominant pH lever in practice (Lointier et al. 2020; Salnikov et al. 2022).

#### Cholesterol and ionic strength

Other important points to be considered to predict how the peptide orients in the bilayer are the membrane fluidity and the medium ionic strength. Cholesterol increases bilayer order and elastic moduli, which suppresses the transient curvature required for toroidal pores and stabilises surface-bound states unless the peptide has sufficient span and amphipathicity. On the other hand, electrolytes screen long-range electrostatics, weakening the initial adsorption of cationic AMPs to anionic membranes and thereby reducing the kinetic advantage of surface binding (Schmidt and Wong 2013; Chakraborty et al. 2020).

## Surface-bound vs. Transmembrane States

Firstly, it is defined the most common states adopted by antimicrobial peptides when they interact with membranes.

### Definition of States

It is well established that certain physicochemical parameters influence peptide–membrane interactions. However, these interactions do not occur uniformly across all peptides. Some peptides adopt a transmembrane orientation, others interact parallel to the membrane surface, and still others occupy intermediate states between surface-bound and fully inserted configurations. It is therefore essential to acknowledge that each physicochemical factor contributes differently, depending on the interaction state.

For example, the average membrane thickness is approximately between 2.6 and 3.1 nm (Fisher et al. 1990). Consequently, for a peptide to achieve full transmembrane insertion, its length must match or exceed this distance. This corresponds to approximately between 16 and 22 amino acid residues in an α-helical conformation (Woolfson 2023) or could be one peptide of the same length in an extended conformation. However, while an extended backbone of the same contour length is conceivable, this state is strongly disfavoured in the hydrophobic core due to unsatisfied backbone hydrogen bonds; hence, stable transmembrane orientations are overwhelmingly helical (Ainavarapu et al. 2007). It is worth noting, however, that the latter scenario is less probable under physiological conditions. In contrast, peptides interacting in a surface-parallel orientation may exhibit virtually any length. Peptides adopting intermediate angles of insertion may have lengths shorter than the membrane thickness without achieving full transmembrane configuration (White and Wimley 1999).

While a minimum peptide length is important for adopting a transmembrane orientation, helix propensity is equally critical. Transmembrane peptides must display an effective hydrophilic/hydrophobic residue partition to properly interact with the bilayer’s hydrophobic core — a partition that typically arises from the formation of a helical structure. In addition, it is common for transmembrane peptides to associate with one another to shield hydrophilic regions from the membrane interior (Krauson et al. 2012). In contrast, peptides that interact in an interfacial or surface-parallel manner are more flexible concerning helix propensity, as the interfacial region of the membrane does not present a sharply defined boundary between hydrophilic and hydrophobic environments (Shai 2002). Nevertheless, helical conformation may still be important for peptides in intermediate insertion states, particularly those approaching transmembrane configurations (Krauson et al. 2012).

If helix propensity is a major factor influencing peptide–membrane interactions, amphipathicity is equally important and is often dependent on the helical structure. Although amphipathicity is important to transmembrane orientation, a helical peptide with perfect well-partitioned hydrophilic and hydrophobic faces is more likely to orient surface-bound into the membrane, as regions with clearly defined polarity align more effectively with the analogous regions of the bilayer. Considering the hydrophobic core of the membrane, only strongly hydrophobic peptide segments can stably insert into this region. Moreover, the peptide’s hydrophobic moment must be oriented toward the membrane core, while the hydrophilic face may interact synergistically with other peptides or with the aqueous environment. In contrast, peptides with poor polar segregation — that is, lacking an amphipathic structure — are more likely to associate superficially with the membrane. This is because their disorganised polarity is more easily accommodated in the interfacial region, where the hydrophilic/hydrophobic boundary is inherently more flexible and less sharply defined (Shai 1999; Pistolesi et al. 2007).

In addition to amphipathicity, side chain steric hindrance and lipid packing must also be considered when analysing peptide–membrane interactions. While aromatic residues are highly hydrophobic and might be expected to interact favourably with the lipid core, their large side chains can significantly disrupt lipid packing when located in the middle of the peptide chain — which globally disfavours membrane insertion. In contrast, when positioned at the termini of the peptide, these same residues can participate in cation–π interactions and help stabilize a transmembrane orientation. Following this rationale, apolar residues with small side chains — such as valine, alanine, or glycine — are better suited to occupy central positions within transmembrane peptides, as they fit more compatibly into the densely packed hydrophobic core of the bilayer. Conversely, the opposite configuration — large or polar residues at central positions — tends to favour surface-parallel peptide–membrane orientations due to less favourable packing interactions (Bechinger 1999; Holt and Killian 2010).

Another factor that may influence peptide orientation is the net charge and its distribution along the peptide chain. In transmembrane peptides that cooperate to form pores—such as in barrel-stave models—the interior of the pore is typically hydrophilic. A high concentration of charges in a localised region may lead to an increased electrostatic potential inside the pore, potentially destabilising it. In contrast, peptides with more evenly distributed charges can reduce this electrostatic buildup and facilitate pore stability. A similar rationale applies to toroidal pores, where distributed charges may interact more effectively with the curved phospholipid headgroups. From this perspective, localised charge clusters may favour surface-bound interactions, while distributed charges may support deeper insertion or pore formation (Chen et al. 2017).

Peptide aggregation may also affect orientation within the membrane. When peptides aggregate in solution, fewer monomeric units are available to interact directly with the membrane. As a result, the peptide–membrane interaction may occur in a more superficial and disorganised manner, reducing the likelihood of forming transmembrane structures or pores. Therefore, higher aggregation tendencies may correlate with surface-bound orientations due to the limited availability of free peptides for organise insertion (Serian et al. 2024).

Conformational dynamics may also play a critical role in determining whether a peptide adopts a surface-bound or transmembrane orientation. Highly dynamic peptides may be unable to form stable, organised structures required for pore formation, favouring instead a surface-bound interaction. This behaviour can be correlated with fluorophore release assays. Peptides that form stable transmembrane pores typically show fixed-rate fluorophore release, controlled by the number and lifetime of pores. In contrast, surface-active peptides often exhibit accelerating release kinetics, rapidly leading to vesicle burst—likely reflecting a disordered, dynamic interaction with the membrane (Bechinger 1999; Wimley and Hristova 2019).

At this point, the distinguishing features that lead to either a transmembrane or a surface-parallel state are identifiable. However, some peptides do not insert fully perpendicular to the membrane, nor do they remain strictly parallel to the surface. Regarding these peptides, a reasonable hypothesis can be made, perhaps they exhibit physicochemical characteristics that favour both interaction modes to some extent. As a result, their behaviour is harder to predict, as they do not follow a canonical interaction pattern. This may be the case for a significant subset of antimicrobial peptides. In summary, peptides may interact with membranes in surface-parallel, fully transmembrane, or partially inserted states, depending on the balance of structural and chemical parameters.

### Thermodynamic considerations

The interaction peptide–membrane, viewed from the thermodynamics perspective, can be sketched from ΔG = ΔH − TΔS. Adsorption of cationic, amphipathic helices to anionic bilayers is typically exothermic, dominated by electrostatics and hydrogen-bonding to headgroups/solvation shells. Insertion adds contributions from peptide/bilayer dehydration and a coil→helix transition ( ≈ − 0.7 to − 0.8 kcal mol⁻¹ res⁻¹), also the hydrophobicity of each amino acid residue, which is quantitatively analysed by the Wimley scale (Wimley and White 1996) count for the insertion. Another important point is the helix dipole, which needs to be oriented in the best fit for the peptide insertion (Hol 1985). This dipole is directly related to the presence of aromatic residues in the helix extremities, where the residues function as an anchor in membrane interface (Johnson et al. 2007). All these processes are often accompanied by entropy gains from the release of ordered water/ions and counterion redistribution. In practice, there is enthalpy–entropy compensation: insertion is not purely “entropically driven” but rather reflects a balance in which dehydration and helix formation can offset orientational/translational restrictions at crowded interfaces (Seelig 2004).

Once the thermodynamic variables have been analysed, it can be said that sustained proteinaceous pores further require oligomer ordering and alignment, incurring translational/rotational entropy costs and, for cationic helices, electrostatic repulsion unless charges are dispersed or screened—one reason many AMPs favour toroidal/lipid-assisted or carpet-like routes over idealised barrel-stave pores. Because these regimes differ thermodynamically, multi-temperature ITC (ΔC_p_) helps distinguish adsorption-dominated profiles from insertion-enabled ones. However, these ITC results need to be paired with ssNMR orientation/tilt to strengthen the assignments (Henriksen and Andresen 2011; Mihajlovic and Lazaridis 2012; Henderson et al. 2016).

## Decision map and worked examples

As a sum of information disponible in the literature, this work aims to collate consistent trends and practical readouts rather than to fit a single mechanistic law to all AMPs. The data bellow is part of this analysis.

Table 1 summarises how physicochemical parameters may influence the orientation of peptides upon interaction with membranes. It is important to note, however, that these outcomes are conceptual predictions and do not necessarily reflect strict or universal behaviour.

**Table 1:** Decision parameters with rationale and threshold ranges

Parameter	Transmembrane-favouring range (with rationale)	Surface-favouring range (with rationale)	Notes
Effective span (nm)	>~2.6–3.1 nm (≈ 16–22 res helix; thicker bilayers require more). Tilt reduces the required N	<~2.6 nm (short helices/unstructured segments)	Hydrophobic mismatch relieved by tilt, local thinning/thickening (Strandberg et al. 2012)
Helix propensity (%)	High; partitioning–folding coupling lowers ΔG upon insertion	Low–moderate; favours interfacial adsorption	Context: lipid composition and temperature shift helicity (Wieprecht et al. 1999)
Amphipathicity (µH)	Clear segregation; µH above typical interfacial thresholds	Poor segregation; mixed polarity tolerates fuzzy interface	Trp/Tyr interfacial anchors assist transmembrane if terminal; central aromatics add steric cost (Yau et al. 1998)
Charge distribution	Distributed/alternating; avoids local electrostatic build-up in pores	Clustered cations; strong interfacial binding; penalizes tight oligomer pores	Ionic strength screens surface adsorption; pH redistributes protonation (His-rich switches) (Lointier et al. 2020)
Aggregation tendency	Low–moderate in bulk; insertion often requires available monomers	High in bulk; reduces effective monomer activity; promotes disordered carpeting	Cooperative pairs (e.g., MAG2/PGLa) are special cases (Harmouche and Bechinger 2018)
Conformational dynamics	Lower (ordered) enables stable pores/transmembrane alignment	Higher (disordered) correlates with carpet/micellization	Cholesterol raises order, can suppress toroidal pores (Chakraborty et al. 2020)

Figure 2 summarises the orientation decision map. The x-axis is effective span (nm), approximated as length × helical fraction × 0.15 nm per residue; the y-axis is conformational order (unitless score, higher = more ordered). Symbols encode aggregation tendency. Points for alamethicin (Yang et al. 2013), aurein 1.2 (Rai and Qian 2017), PGLa (Harmouche and Bechinger 2018), and LL-37 (Lee et al. 2011) illustrate how span and order bias regimes: short/low-order peptides cluster in the interfacial/carpet region; long/high-order sequences fall in tilted/inserted or pore-forming zones; borderline cases shift with pH, anionic fraction, cholesterol and ionic strength. Thus, Fig. 2 is a reading aid to orient expectations and design validation experiments.

### Some worked examples

At this point, it is possible to identify certain peptides that, at least in part, follow the principles proposed by this review. Alamethicin, for instance, is a well-characterised pore-forming peptide that exhibits high helical content and well-defined amphipathicity. It forms highly ordered barrel-stave pores, which implies that the peptide fully inserts into the membrane and displays low conformational dynamics. The peptide effectively spans the bilayer in a perfect α-helical conformation, and it still shares many relevant characteristics, making it a noteworthy reference. This peptide, although predominantly transmembrane, in PE-rich or unsaturated matrix can shift its orientation to in-plane. However, increasing the saturation, a transmembrane pore dominates. Importantly, this review requires validation through experimental data, which may develop over time (Nagao et al. 2015).

Another example worth mentioning is Aurein 1.2, a peptide composed of 13 amino acid residues and characterised by a highly concentrated positive charge. Although it is amphipathic, its charge distribution is not uniform. Aurein 1.2 induces fluorophore release and causes membrane disorganisation and lysis, consistent with a carpet-like or detergent-like mechanism of action. An increase in anionic lipids or a decrease in the cholesterol fraction can cause more fluorophore leakage, and increasing the membrane thickness or cholesterol levels decreases permeabilisation. These features align well with the fact that Aurein 1.2 acts superficially, leading to loss of membrane integrity while maintaining a parallel orientation relative to the bilayer surface (Fernandez et al. 2012).

Another relevant example is the peptide PGLa, which illustrates an intermediate case. PGLa consists of 21 amino acid residues. Additionally, it lacks aromatic residues at its termini, which are often important for anchoring within the membrane interface. However, PGLa displays four well-distributed lysine residues, resulting in a balanced charge distribution that, according to the present study, would favour a transmembrane orientation. What is observed experimentally aligns with this mixed profile: PGLa does not insert fully into the membrane, nor does it remain entirely surface-bound, instead, it adopts a partially inserted configuration. However, his peptide has an alignment that is strongly dependent on lipid saturation, as a saturation increment favours insertion or transmembrane orientation. (Strandberg et al. 2006; Pabst et al. 2008).

These examples and the observations made in this work support the validity of the study and its ability to accommodate non-binary interaction states.

Table 2 summarises some peptide features that can be tracked based on the proposed review.

**Table 2:** Representative antimicrobial peptides: sequence features, dominant orientation regimes, and shift factors

Peptide	~Length/net charge	Dominant orientation/regime (qualitative)	Notes (shift factors)
Magainin-2	23 aa / +3	Mostly surface-bound, changes to toroidal at higher peptide/lipid ratio; strong synergy with PGLa	Saturation increment or pairing with PGLa can promote deeper insertion/ordering (Harmouche and Bechinger 2018)
Melittin	26 aa / +5	Toroidal pores; can adopt transmembrane segments at higher P/L or in ordered matrices	Composition and tension modulate pore stability; kinetic all-or-none permeabilisation reported (Lee et al. 2013)
Indolicidin	13 aa / +4	Predominantly interfacial / carpet-like; disorder and Trp-rich headgroup binding	Strong headgroup interactions; leakage without long-lived proteinaceous pores (Shaw et al. 2006)
Temporin L	13 aa / +2	Surface-bound, membrane-thinning/defect leads to mediated permeabilisation; pore-like states reported	Can induce tubular protrusions/defects; composition and P/L ratio strongly tune activity (Domanov and Kinnunen 2006)
Cecropin A	37 aa / +7	Interfacial alignment with transient insertion during permeabilisation; often toroidal-like	ssNMR shows helices largely parallel at the interface; pores/all-or-none behaviour at thresholds (Marassi et al. 1999)
Piscidin-1	22 aa / +4	Surface-defect / toroidal-favoured; tilted insertion with lipid participation	ssNMR shows tilted, immersed states consistent with lipid-assisted pores (Perrin et al. 2014a)

The review proposed here aims to predict the orientation of antimicrobial peptides based on their physicochemical characteristics. Rather than functioning as a binary classifier, the framework allows for intermediate or ambiguous behaviours, offering a spectrum-based interpretation of peptide–membrane interactions. This flexibility adds to its practical value without compromising its predictive power. However, it is important to acknowledge that the study also has inherent limitations. Therefore, while useful as a guiding framework, the study should be seen as a starting point, open to refinement through further experimental and computational exploration (Shai 1999).

## Experimental approaches

As the study proposed here is a review, its predictions can be experimentally verified using established biophysical techniques. Solid-state NMR can be employed to determine the orientation of peptides within membranes, particularly for sequences of varying lengths, helping to identify whether they adopt transmembrane or surface-bound configurations. These experiments can be performed using either different peptides or variants of a single peptide with systematically altered lengths.

A similar strategy can be applied to evaluate helix propensity. The same peptides whose orientation is determined by solid-state NMR can have their secondary structure assessed using circular dichroism (CD) or solution-state NMR, allowing correlation between helicity and orientation behaviour. Additionally, peptide sequences can be engineered with aromatic residues at their termini, if not already present, to test their effect on membrane insertion and stabilization (Perrin et al. 2014b; Bürck et al. 2016).

Following these structural assessments, fluorophore release assays can be used to evaluate membrane-disruptive activity, distinguishing between pore-forming and carpet-like mechanisms. Typically, constant release rates are associated with stable pore formation, while rapid, accelerating release may indicate surface-level membrane destabilization (Wimley and Hristova 2019; Dutta et al. 2020).

Beyond classic ssNMR/CD/leakage assays, newer helpful approaches can resolve the predicted boundaries of the decision map with higher precision. Neutron reflectometry (NR) quantifies insertion depth/profiles in supported bilayers, while SAXS/SANS with contrast variation detects peptide-induced thinning and curvature in membranes. High-speed AFM on supported bilayers visualizes transient defects or oligomerization in real time, and cryo-EM/cryo-ET captures peptide–lipid assemblies and larger pore architectures. Single-vesicle assays and planar or droplet-interface bilayer electrophysiology resolve stepwise conductance and all-or-none permeabilisation kinetics beyond bulk dye release (Nielsen et al. 2018; Piper et al. 2022).

Finally, molecular dynamics (MD) simulations can complement experimental data by providing atomic-level insights into peptide insertion, aggregation, and lipid reorganization. Together, these experimental and computational approaches can be used to validate and refine the proposed framework (Ulmschneider and Ulmschneider 2018).

## Conclusion

This review offers a valuable tool for predicting the mechanism of action of antimicrobial peptides and guiding their rational design. By correlating measurable parameters—such as peptide length, charge distribution, and helix propensity—with likely interaction states, researchers may be able to design more selective, less toxic, and more bioavailable AMPs. Furthermore, by anticipating whether a peptide is likely to act via pore formation or surface destabilisation, the study can inform strategic choices in drug development, including peptide modifications, delivery strategies, and target membrane profiles. In this way, it contributes not only to theoretical understanding but also to practical applications in antimicrobial drug discovery (Vishnepolsky and Pirtskhalava 2014). The overarching goal is to approach a unified, theory-based model that can explain peptide orientation with as much clarity and accuracy as possible.

Although this work offers valuable insights into peptide–membrane interactions, it does not claim to fully explain all aspects of this complex process. Rather, it provides a conceptual approximation—a theoretical glimpse—grounded in fundamental physical and chemical principles. Importantly, the study is explicitly open to improvement and experimental validation. Even if not fully confirmed, it may still offer a useful route to better understanding the molecular events governing peptide–membrane association. The study remains open to computational refinement as well, including molecular dynamics simulations that can complement experimental approaches. With the core idea now established, this work is ready to be expanded, tested, and integrated into future studies of antimicrobial peptide design and membrane biophysics.

## Data Availability

All data supporting the findings are available within the article.

## Code Availability

The plotting code for Figure 2 is available upon request.
